# Update Review about Metabolic Myopathies

**DOI:** 10.3390/life10040043

**Published:** 2020-04-17

**Authors:** Josef Finsterer

**Affiliations:** Messerli Institute, Krankenanstalt Rudolfstiftung, 1180 Vienna, Austria; fifigs1@yahoo.de; Tel.: +43-1-71165-72085; Fax: +43-1-71165

**Keywords:** metabolism, myopathy, neuromuscular, genetics, mitochondrial deoxy-nucleic acid (mtDNA), fat metabolism, mitochondrial

## Abstract

The aim of this review is to summarize and discuss recent findings and new insights in the etiology and phenotype of metabolic myopathies. The review relies on a systematic literature review of recent publications. Metabolic myopathies are a heterogeneous group of disorders characterized by mostly inherited defects of enzymatic pathways involved in muscle cell metabolism. Metabolic myopathies present with either permanent (fixed) or episodic abnormalities, such as weakness, wasting, exercise-intolerance, myalgia, or an increase of muscle breakdown products (creatine-kinase, myoglobin) during exercise. Though limb and respiratory muscles are most frequently affected, facial, extra-ocular, and axial muscles may be occasionally also involved. Age at onset and prognosis vary considerably. There are multiple disease mechanisms and the pathophysiology is complex. Genes most recently related to metabolic myopathy include *PGM1, GYG1, RBCK1, VMA21, MTO1, KARS*, and *ISCA2*. The number of metabolic myopathies is steadily increasing. There is limited evidence from the literature that could guide diagnosis and treatment of metabolic myopathies. Treatment is limited to mainly non-invasive or invasive symptomatic measures. In conclusion, the field of metabolic myopathies is evolving with the more widespread availability and application of next generation sequencing technologies worldwide. This will broaden the knowledge about pathophysiology and putative therapeutic strategies for this group of neuromuscular disorders.

## 1. Introduction

Metabolic myopathies (MMs) are a heterogeneous group of metabolic disorders characterized by defects of enzymatic pathways involved in myocyte metabolism (inborn errors of metabolism) [1]. MMs can affect the skeletal muscle exclusively, or the muscle plus other organs or tissues (metabolic myopathy plus (MM+), collateral myopathy). In MM+, myopathy may be the dominant feature or a non-dominant collateral feature among others. The limb muscles are predominantly affected in MM, but facial, extraocular, axial, and respiratory muscles may be additionally involved [1]. MMs/MMs+ are heterogeneous hereditary disorders resulting from multiple mechanisms of disease and complex pathophysiologies. Classically, four categories of MMs are differentiated: MMs due to impaired carbohydrate metabolism, MMs due to impaired lipid metabolism, MMs due to impaired energy metabolism, or MMs due to other impaired pathways [2,3]. The most frequently disturbed metabolic pathways in muscle cells leading to MM are mitochondrial pathways, such as the respiratory chain (oxidative phosphorylation), or beta-oxidation, followed by cytoplasmic pathways, including glycolysis (Krebs cycle), glycogen synthesis, or lysosomal pathways, such as glycogen degradation (e.g., Pompe disease) and fat metabolism. During recent years, a number of additional defective pathways have been detected, which may phenotypically manifest as MM/MM+. This review focuses on recently detected abnormal metabolic pathways associated with MM or MM+ and new insights into MMs due to impairment of known established metabolic pathways.

## 2. Clinical Presentation

MMs manifest clinically as episodic conditions or as a permanent, usually progressive disorder. Episodic MMs may manifest during an episode with exercise intolerance, muscle cramps, myalgias, rhabdomyolysis, or myoglobinuria. Between the episodes, patients do not complain about muscle symptoms and the clinical neurologic exam is normal. When the episodes are triggered by short, intensive strain such as weight-lifting, sprinting or a “home run”, the underlying defect is most likely attributable to impaired glycogen metabolism [4]. When the episode is triggered by endurance exercise, such as hiking, biking, football, or even fasting, the underlying defect is most likely attributable to a fatty acid oxidation disorder (FAOD) [4]. Permanent MMs manifest as muscle weakness, muscle wasting, exercise intolerance, easy fatigability, myalgias, sore muscles, muscle cramps, or permanent tiredness. If the respiratory muscles are involved, respiratory distress or respiratory failure may ensue. Mitochondrial disorders (MIDs) frequently manifest with exercise intolerance as initial manifestation. Muscle weakness may rarely lead to orthopedic abnormalities, such as scoliosis, hyperlordosis, or laxity of joints. During exercise, an increase in muscle breakdown products can be observed [1].

## 3. MM due to Impaired Carbohydrate Metabolism

Impaired carbohydrate metabolism may be categorized as impaired glycogen synthesis, impaired glycogen degradation, or impaired glycolysis. MMs due to impaired carbohydrate metabolism are subdivided into MMs with glycogen storage (muscle glycogen storage diseases (GSDs, glycogenoses) with or without the additional deposition of polyglucosan (glycogenoses)), of which 16 are currently known, and into MMs without glycogen storage (aglycogenoses), of which two are currently known. MMs due to impaired carbohydrate metabolism manifest during brief periods of high-intensity exercise, whereas MMs due to defects of lipid metabolism manifest during long-duration, low-intensity endurance exercise or during fasting, surgery or infection [3]. The field of muscle glycogenoses has progressed in recent years by the detection of new subtypes and by achieving a better understanding of the pathophysiology of glycogenoses and the physiology of glycogen metabolism [5].

### 3.1. Glycogen Storage Disorders (GSDs)

GSDs are rare hereditary disorders characterized by the deposition of abnormally structured glycogen in myocytes, and clinically manifesting with exercise intolerance or muscle weakness owing to increased glycogen storage disrupting contractile functions or reduced substrate turnover below the enzymatic block [6,7]. Sixteen different types are currently delineated, of which 11 manifest as MM or MM+ (Table 1). The most common MM due to impaired glycogen metabolism is McArdle’s disease. The only GSD that is treatable is Pompe disease (PD).

#### 3.1.1. AGL (GSD IIIa, Forbes-Cori Disease, OMIM 232400)

The *AGL* gene encodes for the glycogen debrancher enzyme. Mutations in *AGL* result in AGL deficiency, clinically presenting as GSD IIIa. GSD IIIa is an autosomal recessive (AR) disorder clinically characterized by the triad of fasting hypoglycemia, hepatomegaly, and progressive, proximal, vacuolar myopathy with onset in childhood [10]. There is broad phenotypic heterogeneity of *AGL* mutations between different ethnic groups [11]. In a recent study of four Chinese patients with GSD IIIa, the mutations c.206dupA, c.1735  +  1G  >  T and c.2590 C > T were identified as causative [11]. The dominant phenotypic manifestation in these patients was progressive myopathy with weakness and CK-elevation [11]. Additionally, three of the four patients had myocardial thickening, all four patients had hepatomegaly at the initial visit, and one patient had hypoglycemia [11]. In a study of five Iranian patients with GSD IIIa, three patients had typical liver involvement in childhood, and four developed vacuolar myopathy [22]. In a study of 175 patients with GSD IIIa, the most frequent initial manifestation was hepatomegaly [23]. Chronic complications included liver cirrhosis, liver adenoma, or hepatocellular carcinoma in 11%, cardiomyopathy in 15%, heart failure in 58%, myalgia in 34%, and diabetes in 9% of patients [23]. In a study of three patients with GSD IIIa, bulbar muscle involvement with fatty infiltration at the base of the intrinsic tongue musculature has been reported [24].

#### 3.1.2. PYGM (GSD-V, OMIM 232600)

In a recent case report of a 61 year old male with exercise-induced pain since age three, weight loss and creatine-kinase (CK) values up to 4000 U/L and severe axial myopathy with predominant affection of the paraspinal muscles has been described [25].

#### 3.1.3. PGM1 (GSD XIV, OMIM 171900)

The *PGM1* gene encodes for phosphoglucomutase-1, which catalyzes the transfer of a phosphate moiety between glucose one-phosphate and glucose six-phosphate. Mutations in *PGM1* cause GSD type XIV, which is also a congenital disorder of protein N-glycosylation [26]. Patients with GSD XIV present with fixed (permanent), late-onset myopathy with additional involvement of other organs such as the brain, liver, heart, endocrine organs, or the hematopoetic system, manifesting as cognitive impairment, hypoglycemia, hepatopathy, malformations, dilated cardiomyopathy, endocrine deficiencies (adrenal insufficiency, growth retardation, short stature), or hemostatic abnormalities [26,27,28].

#### 3.1.4. GYG1 (GSD XV, OMIM 603942)

The *GYG1* gene encodes for the glycogenin-1 protein (GYG1), the main enzyme responsible for glycogen polymerization [29]. GYG1 is vital for the function of self-glucosylates, using an inter-subunit mechanism to form an oligosaccharide primer which acts as the substrate for glycogen synthase. In addition, GYG1 has a role in regulating glycogen metabolism and the achievement of maximal glycogen levels in skeletal muscle. Mutations in *GYG1* have been recently found to cause late-onset, progressive limb girdle myopathy, also referred to as polyglucosan body myopathy (PGBM) type 2 (Table 1) [20]. However, phenotypes with distal muscle weakness have also been reported, even in the same family [6]. Muscle biopsies show features of a vacuolar myopathy, histochemically and ultrastructurally consistent with PGBM [20]. The heart is usually not involved in these patients [20]. However, the original description of GSD XV included severe cardiomyopathy without PGBM. In another family carrying the compound heterozygous mutations c.484delG and c.403G > A, muscle weakness and wasting were highly variable between family members [6]. The complete absence of GYG1 may only cause a very late-onset asymptomatic myopathy [30]. This indicates that GYG1 is largely disposable for building normal muscle glycogen even without a compensation by GYG2 [31]. Currently, PGBM is associated with mutations in eight different genes, including *GYG1, GBE1, RBCK1 (HOIL-1), PFKM, EPM2A, EPM2B (NHLRC1), PRDM8*, and *PRKAG2* [32]. Polyglucosan is an amylopectin-like polysaccharide derived from defective glycogen metabolism. Unlike glycogen, it is partially resistant to α-amylase digestion [32]. Polyglucosan has a typical fibrillar appearance on electron microscopy and can aggregate into dense inclusions (polyglucosan bodies) [32]. Polyglucosan can be found in association with ageing, but it is also a hallmark of some diseases caused by defective glycogen metabolism [32].

#### 3.1.5. RBCK1 (OMIM 610924)

The *RBCK1* (*HOIL-1*) gene encodes for the E3 ubiquitin ligase, an enzyme that recruits an E2 ubiquitin-conjugating enzyme that has been loaded with ubiquitin, recognizes a protein substrate, and assists or directly catalyzes the transfer of ubiquitin from the E2 to the protein. Mutations in *RBCK1* were first described in three children with failure to thrive, chronic auto-inflammation and recurrent episodes of sepsis [33]. In a subsequent study of 10 patients carrying a *RBCK1* mutation, phenotypic expression included juvenile-onset myopathy and rapidly progressive cardiomyopathy, requiring heart transplantation in four patients [34]. Extensive deposition of polyglucosan was found in myocytes and cardiomyocytes, hence the term PGBM-1 was adopted [34].

## 4. MMs due to Impaired Lipid Metabolism

MMs due to impaired lipid metabolism are a group of recessively inherited disorders due to either the defective import of long-chain fatty acids (LCFAs) into mitochondria (e.g., carnitine cycle disorder) or due to defective mitochondrial beta-oxidation (FAODs). Disorders of lipid metabolism may go along with lipid storage in muscle cells (lipid storage diseases/myopathies (LSMs)) or without intracellular lipid storage [35]. Lipid storage diseases are characterized by the impaired digestion of triglycerides with a consecutive increase in cytosolic lipid droplets [4]. Lipid storage diseases are also characterized by intramuscular lipid accumulation and by impaired mitochondrial bioenergetics in myocytes, causing progressive myopathy with muscle weakness [36]. Typically, triglyceride droplets are excessively stored in muscle tissue in lipid storage diseases [37]. Clinically, lipid storage diseases are characterized by slowly progressive, fixed muscle weakness. Weakness can be proximal in *SLC22A5, ETFDH,* and *CPT-II*-related myopathies. Disorders of lipid metabolism with markedly increased lipid droplets in myocytes include primary carnitine deficiency (PCD), multi-acyl-CoA dehydrogenase deficiency (MADD), and neutral lipid storage disease with myopathy (NLSD-M) (Table 2) [35].

### 4.1. Fatty Acid Uptake Disorders (FAUDs)

The most common of the FAUDs is CPT-II. *CPT2* encodes carnitine palmitoyl-transferase-II, a key enzyme involved in LCFA oxidation [46]. Homozygous mutations in the *CPT2* gene cause CPT-II deficiency, which manifests with three clinical presentations: a lethal neonatal form, a severe infantile form, and a more common and mild myopathic form [41]. The myopathic form is due to missense mutations in the *CPT2* gene, of which the p.Ser113Leu mutation accounts for about 60% of cases [41]. The myopathic form is the most common disorder of lipid metabolism affecting the skeletal muscle but remains frequently undetected [46]. The myopathic form is also the fatty acid uptake disorder that most likely manifests with myoglobinuria. The myopathic form typically presents with exercise intolerance and recurrent attacks of muscle weakness, muscle cramps, and myoglobinuria precipitated by certain drugs (non-steroidal anti-inflammatory drugs (NSAIDs)), prolonged exercise, episodes of fasting, or stress [47]. Males are usually more severely affected than females. Serum CK and serum C12 and C18 acyl-carnitines are elevated during such episodes. Myoglobin is elevated in the urine. CPT-II deficiency can present antenatally as congenital brain malformations [48]. Today, CPT-II patients hardly become symptomatic as they are diagnosed perinatally and receive preventive measures. Treatment is primarily focused on preventing a catabolic state by avoiding triggers such as fasting, vigorous/excessive exercise, ingestion of LCFAs and carnitine, or intake of NSAIDs [41]. Complications may also require appropriate therapeutic measures. Strenuous exercise may induce severe rhabdomyolysis in children and adults with consecutive renal failure in these patients [49]. Although most patients become symptomatic after birth, it has been recently shown that antenatal malformations such as cerebral dysgenesis or neuronal migration defects may be indicative of CPT-II [50].

### 4.2. Fatty Acid Oxidation Disorders (FAODs)

Fatty acid oxidation disorders (FAODs) are caused by impaired beta-oxidation inside the mitochondria or other defects. FAODs are characterized by an episodic course of muscle weakness, stiffness, rhabdomyolysis, or myoglobinuria. The most prevalent of the FAODs are very long chain acyl-coenzyme-A dehydrogenase deficiency (VLCAD) and mitochondrial trifunctional protein deficiency (MTPD) (Table 2). MTPD is due to mutations in the HADHB subunit of the trifunctional enzyme complex, encoded by the genes *HADHA* or *HADHB*. Although pediatric forms are delineated from adult forms (as pediatric forms of FAODs show a more severe course manifesting with MM, hepatopathy, cardiomyopathy and sudden cardiac death (SCD) compared to adult forms, which usually go along only with isolated/pure MM), there is not much difference between them [4]. The severity of FAODs frequently decreases with age. In FAODs, intracellular fat droplets are usually not increased or only mildly increased [35]. Triggers of episodic muscle manifestations include infections, a fat-rich diet, emotional stress, long duration/vigorous exercise, general anesthesia, fasting, or drugs (e.g., ibuprofen, diazepam). Among pediatric patients, clinical manifestations are somehow similar among all types of lipid metabolism disorders, including hypotonia, hypoketotic hypoglycemic encephalopathy, hepatomegaly, and cardiomyopathy [35]. CPT-II may remain underdiagnosed in countries where the disorder is not included in newborn screening programs, such as the UK.

The most recently detected FAOD is ECHS1 deficiency, which is caused by mutations in *ECHS1*. *ECHS1* encodes for short-chain enoyl-CoA hydratase (ECHS1), which is a key fatty acid oxidation enzyme involved in the metabolism of fatty acid acyl-CoA esters [4]. ECHS1 deficiency causes disease; however, the clinical manifestations are unlike most other FAODs [44]. Patients with ECHS1 deficiency commonly manifest with Leigh syndrome, also known as subacute necrotizing encephalomyelopathy, which is usually associated with defects in oxidative phosphorylation [44]. In one patient, complex-IV deficiency has been reported; in another patient, combined complex-I and complex-II deficiency were reported; and in a third patient, isolated complex-III deficiency was reported [44]. In a boy with ECHS1 deficiency, ECHS1 protein expression was severely depleted in the patient’s skeletal muscle and patient-derived myoblasts [45]. A marked decrease in enzyme activity was also evident in patient-derived myoblasts [45].

## 5. Myopathy in Mitochondrial Disorders (MIDs)

MIDs are a heterogeneous group of disorders due to impaired metabolic pathways within mitochondria. The most well-known mitochondrial metabolic pathways involve the respiratory chain, but impairment of a number of other pathways may also go along with MM. Specific MIDs (e.g., mitochondrial encephalopathy, lactic acidosis, and stroke-like episodes (MELAS), myoclonic epilepsy with ragged-red fibers (MERRF), Kearns-Sayre syndrome (KSS), Leigh syndrome, neuropathy, ataxia, and retinitis pigmentosa (NARP), chronic progressive external ophthalmoplegia (CPEO)) are delineated from non-specific MIDs (mitochondrial multiorgan disorder syndrome (MIMODS)) (Table 3). Whether pediatric MIDs are more frequently due to mutations in nDNA located genes and adult-onset MIDs more frequently associated with mtDNA mutations is under debate. Clinical manifestations of MIDs are manifold and in the majority of cases, MIDs manifest as MIMODS frequently involving the muscle [51]. However, a number of MIDs manifesting with isolated MM, particularly at onset of the disease, have been reported. A cardinal feature of mitochondrial myopathy is easy fatigability, which is usually disproportional to the degree of muscle weakness. Frequently, MM in MIDs does not begin with weakness and wasting but with non-specific symptoms, such as myalgia, sore muscles, fatigue, muscle cramps, carpopedal spams, or exercise intolerance. Many patients complain about spontaneous muscle aching and sore muscles without previous muscle exercise. Fixed muscle weakness may first occur in the extraocular eye muscles or the lid elevators, leading to ptosis and external ophthalmoparesis, but all other muscle groups may also be subsequently involved. Myopathy without involvement of the extraocular muscles may additionally occur. Apart from limb muscles, axial and respiratory muscles may also be subsequently involved. Some patients may present with rhabdomyolysis. The number of mutated mitochondrial genes associated with MM or MM+ is rapidly increasing, particularly those from nDNA genes [52]. The most prevalent and recently detected are discussed below.

### 5.1. mtDNA Genes

Most of the genes located on mtDNA can manifest with MM+. However, since other manifestations may prevail, MM is frequently overlooked, particularly at onset of the disease. MM or MM+ in MIDs due to mutations of mtDNA are well appreciated and are thus not discussed in detail here.

### 5.2. nDNA Genes

#### 5.2.1. ISCA2

An example for a novel cause of subclinical non-dominant MM+ is a mutation in the *ISCA2* gene. The *ISCA2* gene encodes for the iron–sulfur cluster (ISC) assembly-2 protein. Together with other enzymes, ISCA2 is essential for the maturation of ISCs. The assembly of ISCs is a vital cellular process for the generation of ISC-containing proteins, which are involved in the regulation of gene expression, respiration, energy production by means of the respiratory chain, sensing of oxygen, and cellular iron concentration [52]. So far, 19 patients carrying *ISCA2* mutations have been reported, which predominantly phenotypically manifested with early onset central nervous system abnormalities [52]. However, other studies have documented that the muscle may be clinically or sub-clinically affected. Muscle biopsy may show increased fiber size variability, increased fiber atrophy and reduced respiratory chain activity in some of these patients [61].

#### 5.2.2. KARS

The *KARS* gene encodes for a mitochondrial and cytoplasmic lysyl-tRNA synthetase, phenotypically manifesting as AR Charcot-Mary-Tooth (CMT) polyneuropathy, as AR non-syndromic hypoacusis, or as congenital visual impairment and microcephaly [62]. Recently, a 14 year old female with cardiomyopathy, psychomotor delay, and mild myopathy with combined complex-I and complex-IV-defect on biochemical investigations of the muscle homogenate has been reported. Complex-I activity was reduced by 45% and complex-IV activity was reduced by 72% [62]. Muscle histochemistry revealed marked and diffuse reduction of cytochrome-c-oxidase (COX) staining in all muscle fibers. The patient carried two novel mutations in the *KARS* gene [62].

#### 5.2.3. TK2

The thymidine-kinase-2 gene on chromosome 16 encodes for thymidine-kinase-2, which converts deoxy-pyrimidine nucleosides to nucleotides by phosphorylation, which is the first step to generate the dTTP and dCTP building blocks required for mtDNA synthesis [63]. Mutations in *TK2* secondarily cause mtDNA depletion. Clinically, *TK2* mutations manifest with infantile-onset myopathy, predominantly affecting the proximal limb muscles (Duchenne-like, or limb-girdle muscular dystrophy (LGMD)-like presentations) and progressing to muscular respiratory failure due to involvement of the respiratory muscles [63]. Recently, 82 patients with myopathy due to *TK2*-mutations have been reviewed [63]. Although myopathy was the prominent phenotypic feature in all patients, some patients additionally presented with cerebral involvement, ocular involvement, or liver involvement [63].

#### 5.2.4. MTO1

Mutations in the mitochondrial tRNA translation optimisation-1 (*MTO1*) gene, encoding an evolutionary conserved protein expressed in high-energy demand tissues, result in a phenotype characterized by global developmental delay, intellectual disability, seizures, ataxia, failure to thrive, feeding difficulties, generalized hypotonia, optic atrophy, lactic acidosis, hypertrophic cardiomyopathy, and complex-I and complex-IV deficiency in muscle tissues [64]. According to a recent retrospective analysis of 35 patients carrying a *MTO1* mutation, a small number of patients with myopathy of the skeletal muscles has been reported [64]. *MTO1* deficiency is lethal in some cases. Patients with complete loss of *MTO1* are not viable [64]. Subjective clinical improvement has been observed in a small number of patients on therapies such as the ketogenic diet or dichloro-acetate [64].

#### 5.2.5. Coenzyme-Q Deficiency and Myopathy

Coenzyme-Q deficiency is due to mutations in >10 genes. Coenzyme-Q not only functions as an electron donor and acceptor within the respiratory chain, but also performs several other functions in cellular metabolism [65]. For example, coenzyme-Q functions as an electron carrier in the first step of the hydrogen sulfide oxidation pathway in which sulfide quinone oxidoreductase (SQR) oxidizes sulfide to sulfite, and where coenzyme-Q functions as an electron donor [65]. Recent studies have shown that coenzyme-Q deficiency also results in a reduction of SQR levels [65]. Only a few patients develop myopathy, but more frequently ataxia and renal insufficiency are seen.

## 6. Others

### 6.1. Lysosomal Storage Disease (LSD)

LSDs are a heterogeneous group of rare, inherited metabolic diseases that are commonly triggered by the accumulation of lipids or glycogen inside organelles of the endosomal–autophagic–lysosomal system (EALS) [66]. Disrupted lysosomal homeostasis (i.e., lysosomal cacostasis) contributes to common neurodegenerative disorders such as Parkinson’s disease, amyotrophic lateral sclerosis (ALS), CMT, hereditary spastic paraplegias (HSPs), or multiple systemic atrophy (MSA) (Table 4) [66]. Lysosomal disorders manifesting with MM include Pompe disease and Danon disease.

#### 6.1.1. GAA (GSD II (Pompe Disease, OMIM 606800))

Pompe disease (PD) is a lysosomal storage disease (LSD) caused by mutations in *GAA* which encodes the enzyme acid maltase (alpha 1,4-glucosidase) [9]. Acid maltase is a lysosomal enzyme responsible for the breakdown of glycogen into glucose. PD is characterized by glycogen storage in lysosomes and the cytoplasm of myocytes, cardiomyocytes, and other cell types [9]. An early onset type (EOPD) and an adult-onset type (LOPD) are delineated [9]. PD follows an AR trait of inheritance.

##### EOPD

EOPD is clinically characterized by failure to thrive, dysphagia, myopathy, and severe hypertrophic cardiomyopathy leading to heart failure [71]. If untreated, EOPD has a poor outcome with death before one year of age [71]. Hypotonia and cardiomyopathy often lead to a dry blood spot (DBS) test, and if positive, to *GAA* sequencing. Muscle biopsy with ultrastructural investigations, as previously proposed [9], is usually not the first choice to diagnose the condition. In some patients, myopathy is only an ancillary manifestation in EOPD.

##### LOPD

LOPD is characterized by progressive muscle weakness of the proximal limb, axial, and respiratory muscles [71]. Patients with LOPD present with proximal limb weakness resulting in gait disturbance with a positive Trendelenburg and Gower’s sign, scapula alata, wasting of the paraspinal muscles, myalgias, and falls. Involvement of the respiratory muscles may be associated with sleep-disordered breathing together with hypoventilation, dyspnoea, and obstructive sleep apnea syndrome (OSAS) [71]. Progressive dysfunction of the voice apparatus in LOPD has been reported in one study [71]. Severe cardiomyopathy can occasionally be the initial manifestation of LOPD [71]. Involvement of the peripheral nerves may lead to polyneuropathy. Only few patients with LOPD may have normal GAA activity [72]. LOPD is usually less progressive than EOPD if untreated. To assess the risk of relatives of an index patient, enzymology (GAA activity assay) is recommended as the initial screening test before targeted mutation analysis is carried out [73,74]. In a recent study of four patients with LOPD, fluoro-desoxy-glucose positron-emission tomography (FDG-PET) failed to demonstrate a correlation between glucose uptake and abnormal glucose storage [74]. Muscle MRI disclosed the extent of muscle involvement and specific MRI sequences allowed differentiation between muscle edema and muscle atrophy [75]. Genotype/phenotype correlations have been recently presented in a study of 1079 patients from Pompe registry sites [76].

#### 6.1.2. LAMP2 (Danon Disease)

The lysosome-associated membrane protein-2 (*LAMP2)* encodes for a lysosome-associated membrane glycoprotein. There are three isoforms, LAMP2A, LAMP2B, and LAMP2C, of which LAMP2B is the major isoform expressed in muscle tissue [77]. LAMP2 is required for the maturation of autophagosomes by fusion with lysosomes. Thus, LAMP2 deficiency leads to failure and disturbance of macroautophagy [78]. Mutations in *LAMP2* manifest phenotypically with the characteristic triad of fixed, progressive, proximal myopathy, cardiomyopathy, and intellectual disability with onset in adolescence [67]. Myopathy in Danon disease may show a fluctuating course and may be easily misdiagnosed until a muscle biopsy shows typical vacuolar myopathy [79]. A striking morphological feature on muscle biopsy are small basophilic granules, which are in fact autophagic vacuoles surrounded by sarcolemma-like membranes [77]. Recently, it has been shown that differentiating vacuolar myopathy due to *LAMP2* variants from other types of vacuolar myopathy can be facilitated by application of p62, microtubule-associated protein 1A/1B-light chain 3 (LC-3), and LAMP2 staining [80]. According to a recent study, mental retardation is a core constituent of Danon disease, which is why these patients need to be referred to a neuropsychologist or psychiatrist [81]. Cardiomyopathy in *LAMP2* carriers may be associated with conduction defects, such as Wolf–Parkinson–White syndrome, which may be complicated by cardiac arrest secondarily leading to ischemic stroke [82]. Cardiomyopathy may also be complicated by therapy-resistant heart failure, leading to some of these patients requiring heart transplantation [67]. Due to the rarity of the disease, there is little understanding of the genotype–phenotype correlation and the broad phenotypic heterogeneity of the disease [83]. Though Danon disease is an X-linked disorder, female carriers of the disease may be also affected, particularly in the heart [84]. Rarely, Danon disease manifests without myopathy or mental impairment but exclusively with cardiomyopathy [85,86].

#### 6.1.3. VMA21

*VMA21* encodes for the master assembly chaperone of the vacuolar H+-ATPase (V-ATPase), which is a multi-subunit proton pump for acidifying organelles and thus is vital to all mammalian tissues [68]. Mutations in *VMA21* manifest exclusively as adult-onset vacuolar myopathy, but interestingly, asymptomatic autophagic vacuolar changes in extra-muscular tissue, such as the myocardium, have been recently reported [68]. Irrespective of the mutations detected so far, myopathy is the exclusive manifestation of *VMA21* mutation carriers, and since *VMA21* is located on the X-chromosome, it occurs only in males (X-linked myopathy with excessive autophagy (XMEA)) [68]. XMEA usually progresses to the respiratory muscles, which is why patients become ventilator dependent over time [68].

## 7. Diagnosis

### 7.1. Carbohydrate Metabolism

The diagnosis of GSDs is based on clinical presentation, blood chemical investigations, needle electromyography, the rarely used forearm ischemic exercise test, muscle biopsy, biochemical investigations, and the genetic work-up. The forearm ischemic exercise test relies on the determination of lactate and ammonia under ischemic conditions. Absence of an increase of serum lactate after resolution of ischemia is indicative of a GSD [4]. In McArdle’s disease, CK is permanently elevated, but it is only transiently elevated in phosphofructo-kinase deficiency.

### 7.2. Lipid Metabolism

The diagnosis of FAODs and fatty acid uptake disorders is based on newborn screening programs (LC-MS/MS based acyl-carnitine profiling), clinical presentation, blood chemical investigations (acyl-carnitine profile, amino acids), urine analysis (urine organic acids, ketones, dicarboxylic acids, 3-methyl-glutaconic acid), tandem mass spectroscopy (measures acyl-carnitines C12-C18), needle electromyography, muscle biopsy, and genetic investigations. Urine organic acid analysis can reveal dicarboxylic aciduria in the beta-oxidation defects and the absence of ketones in most of the fat oxidation defects. Elevated 3-methylglutaconic acid is found in MIDs and Barth syndrome. Acyl-carnitines are used to identify all FAODs in the first instance. Acyl-carnitines are elevated in CPT-II, VLCAD deficiency, trifunctional protein deficiency, medium-chain acyl-CoA dehydrogenase (MCAD), and short-chain acyl-CoA dehydrogenase (SCAD) deficiency. Reduced acyl-carnitines are usually dietary or found in severe cases of PCD. Enzyme testing and gene studies are offered, particularly for VLCAD. A FAOD can be suspected in cases of Reye syndrome (hepatopathy plus encephalopathy), impaired consciousness, myopathy, rhabdomyolysis, myoglobinuria, cardiomyopathy, ventricular arrhythmias, or SCD. Hypoglycemia and increased muscle enzymes, ammonia, lactate, myoglobin, or carnitine are indicative of a FAOD. In the urine, organic acids, acyl-carnitine, acyl-glycine, or myoglobin can be detected. Elevated serum carnitine is indicative of CPT-II deficiency and decreased serum carnitine is indicative of PCD. Myopathy due to VLCAD deficiency can be diagnosed upon immuno-histochemical investigations of the muscle biopsy. CPT-II deficiency may be underdiagnosed. CK in FAUDs may be normal outside of episodes of rhabdomyolysis. For all FAODs, genetic testing is available. Enzyme testing is available for most FAODs and for CPT-I and CPT-II. Carnitines may be elevated even between episodes.

### 7.3. Mitochondrial Disorders

The diagnosis of MIDs is based on clinical presentation, resting serum lactate and pyruvate, the lactate stress test, needle electromyography (EMG), muscle MRI, magnetic resonance (MR)-spectroscopy, muscle biopsy (including histological, immuno-histological, ultrastructural, biochemical, and polarographic investigations), and a genetic work-up. The extraction of oxygen from blood is reduced in MIDs. Normal muscle biopsy findings do not exclude the presence of a mitochondrial disorder (MID). There is increasing evidence that whole exome sequencing (WES) is the method of choice for diagnosing conditions such as CoQ-deficiency [87] or MIDs in general [88]. The analysis of mtDNA from urine epithelial cells or muscle is helpful.

## 8. Treatment

Treatment of MMs is most frequently symptomatic and only rarely causative. Symptomatic treatment may be non-invasive or invasive. Non-invasive symptomatic treatment includes physiotherapy, diet, application of drugs, conservative orthopedic measures, and non-invasive ventilation. The avoidance of triggers for episodic forms of MM is also important. Invasive measures include orthopedic surgery and invasive mechanical ventilation.

### 8.1. Carbohydrate Metabolism

Ample evidence has been collected during recent years that exercise training in the various types of GSD can be beneficial regarding exercise tolerance and increasing fatty acid oxidation [7]. Oral galactose treatment may have a beneficial effect for some of the clinical manifestations in PGM1-associated GSD [26]. However, in a recent study, it has been shown that standard dosages of 1–2.5 g/kg body weight (BW) are not sufficient to restore impaired glycosylation in all patients [89]. For GSD III, a high protein diet has been reported to improve cardiomyopathy [90]. Recently, it has been shown that albuterol could be an adjunctive therapy for patients with EOPD, although the overall benefit seems to be limited [91].

### 8.2. Lipid Metabolism

Fat metabolism disorders should be generally treated with a carbohydrate-rich diet to avoid a catabolic state [92,93]. Replacement of long-chain fatty acids by medium-long chain fatty acids can be beneficial for VLCAD deficiency. Carnitine is effective in treating PCD and MADD. Riboflavin or carnitine is usually beneficial in MADD [36]. Bezafibrate has not been shown to be effective in vivo [92]. Triheptanoin and trioctanoin have been shown promising results in some FAODs.

### 8.3. Mitochondrial Disorders

The treatment of MIDs relies on non-invasive or invasive measures. Non-invasive symptomatic measures include drugs such as antioxidants, electron donors, electron acceptors, alternative energy resources, or co-factors. Coenzyme-Q is particularly effective in treating primary coenzyme-Q deficiency. An important measure of treatment is the avoidance of mitochondrion-toxic agents. In case respiratory muscles are involved, invasive or non-invasive artificial ventilation is indicated [94]. Treatment of specific aspects such as stroke-like episodes is based on the application of anti-seizure drugs or L-arginine. Deoxynucleoside therapy can be beneficial for TK2-related myopathy.

### 8.4. Lysosomal Storage Diseases

GSD II is the only MM for which an effective therapy is available. It is an enzyme replacement therapy (ERT) in which a recombinant human alpha glucosidase (rhGAA; 20 mg/kg intravenously every two weeks) is applied [95]. The earlier ERT is initiated, the more effective it is. A number of studies using rhGAA alone or in combination with chaperones are currently ongoing. 

## 9. Conclusions

The number of known metabolic myopathies is steadily increasing. Mutated genes most recently identified that cause metabolic myopathy include *PGM1, GYG1, RBCK1, VMA21, MTO1, KARS,* and *ISCA2*. Myopathy may occur in a non-syndromic setting (isolated), but most frequently, myopathy is a collateral feature of the phenotype. In multisystem disease, MM+ may be masked by more prevailing phenotypic features. Phenotypes with collateral myopathy may show broad inter- and intra-familial phenotypic heterogeneity, even if the phenotype is due to the same mutation. Thus, the genotype–phenotype correlation is often poor and larger cohorts than those previously investigated need to be studied. Concerning the optimal diagnostic pathway for patients, it becomes increasingly evident that WES is the method of choice to diagnose coenzyme-Q deficiency and MIDs in particular.

## Figures and Tables

**Table 1:** Mutated nuclear DNA (nDNA) genes leading to defective carbohydrate metabolism and their association with metabolic myopathies (MM) or MM+. GSD = glycogen storage disease, PGBM = polyglucosan body myopathy.

Gene	Pathway	MM	MM+	Reference
*GYS2*	GSD 0	Yes	Yes (cardiomyopathy)	[8]
*GAA*	GSD II	Yes	Yes (cardiomyopathy, polyneuropathy)	[9]
*AGL*	GSD III	No	Yes (hepatopathy, cardiomyopathy, seizures, retardation)	[10,11]
*GBE1*	GSD IV	No	Yes (cardiomyopathy, hepatopathy)	[12]
*PYGM*	GSD V	No	Yes (retinopathy)	[13]
*PFK*	GSD VII	No	Yes (floppy infant, hemolysis)	[14]
*PGAM2*	GSD X	No	No	[15]
*ALDOA*	GSD XII	No	Yes (anemia, hyperkalemia)	[16]
*ENO-3*	GSD XIII	Yes	No	[17]
*PGM1*	GSD XIV	No	Yes (hypoglycaemia, hepatopathy)	[18,19]
*GYG1*	GSD XV (PGBM-2)	Yes	Yes (cardiomyopathy)	[20]
*RBCK1*	PGBM-1	Yes	Yes (cardiomyopathy, infections)	[21]

**Table 2:** Mutated genes associated with lipid metabolism disorders and MM or MM+.

Gene	Location	Pathway	MM	MM+	Reference
*SLC22A5*	PCD	FA import	Yes	Yes (cardiomyopathy)	[38]
*ETFDH*	MADD	FAOD	Yes	Yes (skin disease, hepatopathy, hypoglycemia)	[39]
*PNPLA2*	NLSD-M	ATGL ↓	No	Yes (cardiomyopathy)	[35,40]
*CPT2*	CPT-II	FA import	Yes	Yes (encephalopathy)	[41]
*HADHB*	MTPD	Beta-oxidation	No	Yes (cardiomyopathy, neuropathy)	[42]
*ACADVL*	VLCADD	Beta-oxidation	No	Yes (cardiomyopathy, retardation, hepatopathy)	[43]
*ECHS1*	FAOD	Beta-oxidation	No	Yes (seizures, dystonia, developmental delay)	[44,45]

MADD: multiple acyl-CoA dehydrogenase deficiency (carnitine or riboflavin sensitive), PCD: primary carnitine deficiency (carnitine sensitive), FA: fatty acid import, NLSD-M: neutral lipid storage disease with myopathy, ATGL: adipose triglyceride lipase, MTPD: mitochondrial trifunctional protein deficiency.

**Table 3:** Mutated nDNA genes leading to mitochondrial dysfunction associated with MM or MM+ (only a small sample of mitochondrial myopathies is depicted).

Gene	Location	Pathway	MM	MM+	Reference
tRNA(Leu)	mtDNA	RC	No	Yes	[53]
tRNA(Lys)	mtDNA	RC	No	Yes	[54]
mtDNAΔ	mtDNA	RC	No	Yes	[55]
*MPV17*	nDNA	mtDNA depletion	No	Yes (hepatopathy, encephalopathy)	[56]
*NDUFB8*	nDNA	complex-I ↓	No	Yes (parkinsonism)	[57]
*SLC25A1*	nDNA	complex-V ↓	No	Yes (cognitive impairment)	[58]
*HSPB8*	nDNA	autophagy	No	Yes (neuropathy)	[59]
*MRM2*	nDNA	mt rRNA	No	Yes (MELAS-like)	[60]

RC: respiratory chain.

**Table 4:** Mutated nDNA genes leading to dysfunction of other pathways than those previously descirbed and their association with MM or MM+.

Gene	Pathway	MM	MM+	Reference
*LAMP2*	Lysosome	No	Yes (cardiomyopathy)	[67]
*VMA21*	Proton pump	Yes	No (liver disease)	[68]
*AHCY*	Purine	No	Yes (encephalopathy, hypotonia)	[69]
*MADA*	Purine	Yes	Yes (cardiomyopathy)	[70]

## Nomenclature

**List of Abbreviations**	
AD	Autosomal dominant
AR	Autosomal recessive
CPT-II	Carnitine-palmitoyl-transferase deficiency
DBS	Dried blood spot test
EALS	Endosomal autophagic lysosomal system
EOPD	Early onset Pompe disease
ERT	Enzyme replacement therapy
FAOD	Fatty acid oxidation disorder
FAUD	Fatty acid uptake disorder
GSD	Glycogen storage disease
HCMP	Hypertrophic cardiomyopathy
ISC	Iron–sulfur cluster
LCFA	Long chain fatty acids
LOPD	Late onset Pompe disease
LSD/M	Lysosomal storage disease/myopathy
MADD	Multiple acyl-CoA dehydrogenase deficiency
MID	Mitochondrial disorder
MIMOD	Mitochondrial multiorgan disorder syndrome
MM	Metabolic myopathy
MM+	Metabolic myopathy plus organs other than the muscle are additionally affected
MTPD	Mitochondrial trifunctional protein deficiency
NLSD-M	Neutral lipid storage disease with myopathy
NSAIDs	Non-steroidal anti-inflammatory drugs
OSAS	Obstructive sleep apnea syndrome
PGBM	Polyglucosan body myopathy
PCD	Primary carnitine deficiency
PD	Pompe disease
PEO	Progressive external ophthalmoplegia
SCD	Sudden cardiac death
VLCAD	Very long chain acyl-CoA dehydrogenase deficiency
WES	Whole exome sequencing
XMEA	X-linked myopathy with excessive autophagy
**List of Gene Names**	
*ACADVL*	Acyl-CoA Dehydrogenase Very Long Chain
*AGL*	Amylo-Alpha-1,6-Glucosidase
*AHCY*	Adenosylhomocysteinase
*ALDOA*	Aldolase
*CPT2*	Carnitine Palmitoyltransferase-2
*ECHS1*	Enoyl-CoA Hydratase, Short Chain 1
*ENO3*	Enolase 3
*EPM2A*	EPM2A Glucan Phosphatase, Laforin
*ETFDH*	Electron Transfer Flavoprotein Dehydrogenase
*GAA*	Glucosidase Alpha, Acid
*GBE1*	1,4-Alpha-Glucan Branching Enzyme 1
*GYG1*	Glycogenin 1
*GYS2*	Glycogen Synthase 2
*HADHB*	Hydroxyacyl-CoA Dehydrogenase Trifunctional Multienzyme
*HSPB8*	Heat Shock Protein Family B (Small) Member 8
*ISCA2*	Iron–Sulfur Cluster Assembly 2
*KARS*	Lysyl-tRNA Synthetase 1
*LAMP2*	Lysosomal Associated Membrane Protein 2
*MADA*	Myoadenylate-deaminase
*MPV17*	Mitochondrial Inner Membrane Protein MPV17
*MRM2*	Mitochondrial RRNA Methyltransferase 2
*MTO1*	Mitochondrial tRNA Translation Optimization 1
*NDUFP8*	NADH:Ubiquinone Oxidoreductase Subunit B8
*PFKM*	Phosphofructokinase, Muscle
*PGAM2*	Phosphoglycerate Mutase 2
*PGM1*	Phosphoglucomutase 1
*PNPLA2*	Patatin-Like Phospholipase Domain Containing 2
*PRDM8*	PR/SET Domain 8
*PRKAG2*	Protein Kinase AMP-Activated Non-Catalytic Subunit Gamma 2
*PYGM*	Glycogen Phosphorylase, Muscle Associated
*RBCK1*	RANBP2-Type and C3HC4-Type Zinc Finger Containing 1
*SLC22A5*	Solute Carrier Family 22 Member 5
*SLC25A1*	Solute Carrier Family 25 Member 1
*TK2*	Thymidine Kinase 2
*VMA21*	Vacuolar ATPase Assembly Factor VMA21
